# CO_2_ Angiography in the Standard and Complex Endovascular Repair of the Abdominal Aorta—A Narrative Review of the Literature

**DOI:** 10.3390/jcm13164634

**Published:** 2024-08-07

**Authors:** Paolo Spath, Stefania Caputo, Federica Campana, Enrico Gallitto, Rodolfo Pini, Chiara Mascoli, Andrea Vacirca, Gianluca Faggioli, Mauro Gargiulo

**Affiliations:** 1 Vascular Surgery, University of Bologna, DIMEC, 40138 Bologna, Italy; caputostefania@hotmail.it (S.C.); campanafederica4@gmail.com (F.C.); enrico.gallitto@gmail.com (E.G.); rudypini@gmail.com (R.P.); gianluca.faggioli@unibo.it (G.F.); 2 Vascular Surgery Unit, Hospital «Infermi», AUSL Romagna, 47923 Rimini, Italy; 3 Bologna Vascular Surgery Unit, IRCCS University Hospital S. Orsola, 40138 Bologna, Italy; chiara.ma@yahoo.it (C.M.); andrea.vacirca88@hotmail.it (A.V.)

**Keywords:** carbon dioxide angiography, endovascular aneurysm repair, fenestrated endovascular repair, branched endovascular repair, thoracic endovascular repair, renal function protection, contrast-induced renal injury

## Abstract

**Background/Objectives**: Carbon dioxide digital-subtraction angiography (CO_2_-DSA) is an increasingly adopted technique in endovascular aortic repair (EVAR) and fenestrated/branched EVAR (F/B-EVAR); it is used to reduce the amount of iodinate contrast medium (ICM) and prevent postoperative renal function worsening (PO-RFW). Our aim is to report results from the literature on EVAR and F/B-EVAR procedures using CO_2_-DSA, together with wider applications in aortic endovascular treatment. **Methods**: We performed a literature review by searching electronic databases for published data on CO_2_-DSA during EVAR and F/B-EVAR procedures. The endpoints were postoperative renal function worsening (PO-RFW) and efficacy of intraoperative arterial visualization. Further, applications of CO_2_ for thoracic endovascular aortic repair (TEVAR) were described. **Results**: Seventeen studies reporting results on CO_2_-DSA in EVAR (644 patients) were retrieved. Overall, 372 (58%) procedures were performed with CO_2_ alone, and 272 (42%) were performed with CO_2_+ICM. Eight studies analyzed the effect of CO_2_-DSA angiography on PO-RFW; four studies showed a significantly lower rate of PO-RFW compared to ICM. Five studies (153 patients) analyzed intraoperative arterial visualization with CO_2_-DSA; renal and hypogastric arteries were effectively visualized in 69% and 99% of cases, respectively. The use of CO_2_-DSA in F/B-EVAR has not been widely investigated. The largest series reported that PO-RFW was lower in the CO_2_ vs. ICM group. **Conclusions**: Carbon dioxide is widely applied in modern aortic endovascular treatment. CO_2_-DSA for EVAR and F/B-EVAR is an efficient technique for reducing PO-RFW while allowing acceptable arterial intraoperative visualization.

## 1. Introduction

Endovascular aortic repair (EVAR) often represents the first choice of treatment for aortic pathologies, both in the abdominal (AAA) and thoracic segment, having lower early morbidity and mortality rates [1,2] than open repair [3,4] and being patients’ preference. At the same time, due to its wider application, other issues directly connected to the nature of the endovascular repair might be common and need be managed and prevented in EVAR patients [5]. 

Acute kidney injury (AKI) represents one of the most frequent potential postoperative complications in patients treated for abdominal aortic aneurysm (AAA) with EVAR, impacting up to 20% of cases [6]. Specifically, nephrotoxicity secondary to intraprocedural use of iodinated contrast medium (ICM) represents the most frequent cause of postoperative acute kidney injury (AKI) [7], especially in patients with chronic kidney disease (CKD). This complication has been recently defined in the literature as post-contrast acute kidney injury (PC-AKI), affecting 4% to 18% of EVAR cases depending on the volume of ICM employed during the procedure and the preoperative hydration protocols used [6,8,9,10,11]. 

To minimize the use of ICM, carbon dioxide (CO_2_) has been proposed as alternative contrast medium during digital subtraction angiographies (DSA). CO_2_-DSA’s advantages and disadvantages are summarized in Table 1. Carbon dioxide’s physical and chemical characteristics include low viscosity, high solubility, and fast dissolution in blood (21 times higher than nitrogen, with which air is rich) [12]. CO_2_’s high solubility allows its injection into the arteries below the diaphragm without any clinically significant gas embolism [12]. However, CO_2_ injection above the diaphragm is prohibited since it can be neurotoxic, causing disruption of the blood–brain barrier, seizures, and loss of consciousness [13,14]. 

**Figure 1:**
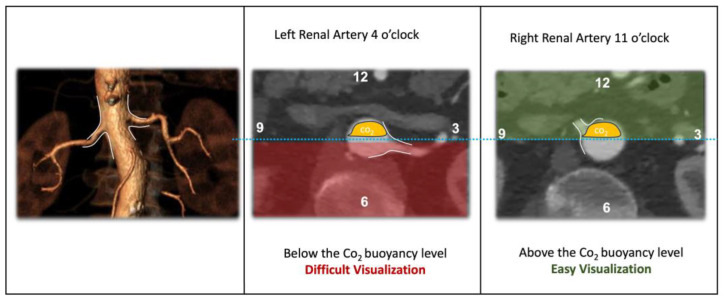
Schematic representation of the CO_2_ buoyancy effect and its impact on arterial visualization. CO_2_ places itself at the upper level of the aorta; therefore, vessels originating from the posterior wall (from clock time 3 to 9), as in this example of the left renal artery, will be difficult to detect; on the other hand, vessels originating from the anterior wall of the aorta (from clock time 9 to 3) will be easily detected, as in the right renal artery shown in this example.

Carbon dioxide’s pathognomonic physical characteristic is its high buoyancy, meaning its ability to float in blood because the upwards force pushing up the fluid is significantly greater than the weight of CO_2_ [15]. This can be highlighted in the cross-table lateral projection of large diameter vessels such as the aorta, and it is responsible of the challenging visualization of visceral vessels originating from the posterior aortic wall, such as posterior renal arteries, which might represent a limitation of aortic endovascular repair in specific anatomies (Figure 1). 

**Figure 2:**
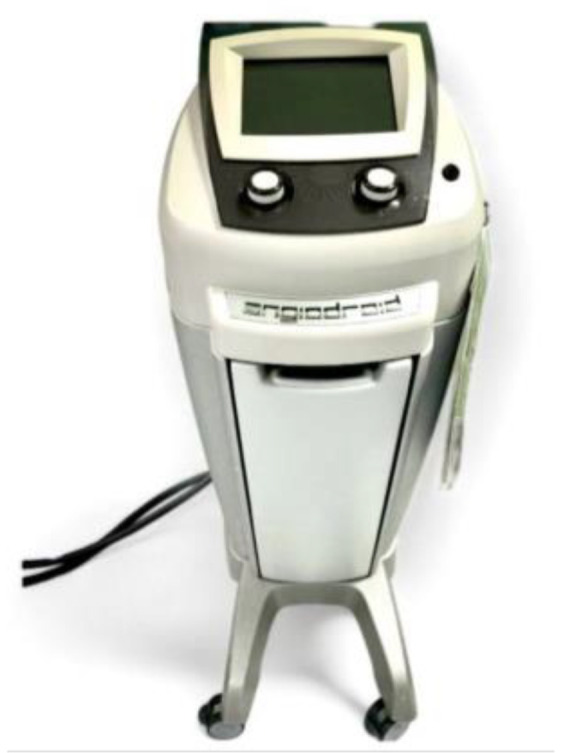
Angiodroid automated injector (Angiodroid S.p.A., San Lazzaro di Savena, Italy).

CO_2_-DSA can be performed manually or by automatic injection systems. Manual injection is conducted using a CO_2_ cylinder/laparoscopic insufflator with an inline bacterial filter, while the automatic injector, with the most widespread in Europe being the Angiodroid injector (Angiodroid S.p.A., San Lazzaro di Savena, Italy) (Figure 2), is a mobile, computerized injector with a dedicated remote control system that allows for setting specific injection volumes and pressures. Its advantages include reduced operator radiation exposure for operators and the ability to precisely control injection volumes and pressure, thus potentially reducing associated side effects and eliminating the risk of air contamination.

Over the years, CO_2_DSA angiographies have been increasingly adopted in endovascular peripheral [16] and aortic repair [14,17,18] alone or with ICM.

However, because at the present time, the use of CO_2_ in endovascular aortic procedures lacks strong evidence, CO_2_-DSA as an alternative to ICM for PC-AKI prevention was not present in the previous version and has still not been mentioned in the most recent European Society for Vascular Surgery (ESVS) 2024 Clinical Practice Guidelines on the Management of Abdominal Aorto-Iliac Artery Aneurysms [1,5].

The aim of this paper is therefore to report the current state of the art of the use of CO_2_ in endovascular aortic repair and to perform a literature review reporting the currently available outcomes of CO_2_-DSA in both standard and complex endovascular repair. 

## 2. Materials and Methods

**Figure 3:**
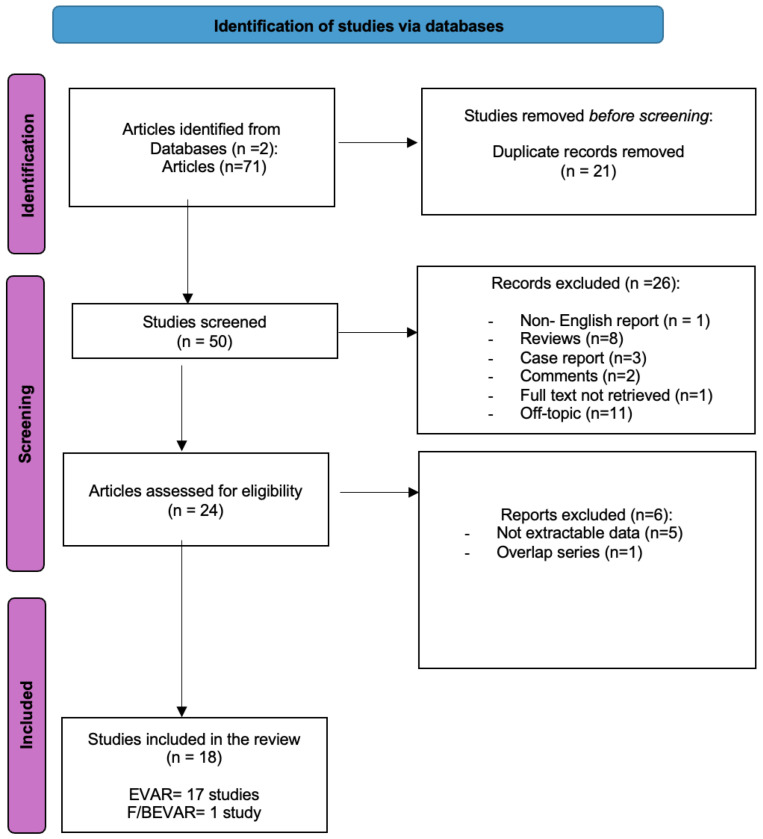
Flowchart of the selection process. EVAR = endovascular aortic repair; F/BEVAR = fenestrated/branched EVAR.

A literature search of two electronic databases (PubMed, Scopus Library) was performed to identify all published papers reporting on the use of CO_2_-DSA in endovascular aortic treatments. We used the following key words: (carbon dioxide) AND (angiography) AND (endovascular aortic repair) (Figure 3). Our literature search was performed independently by two authors (PS and SC), while data extraction was performed by one author alone (FC). 

**Figure 4:**
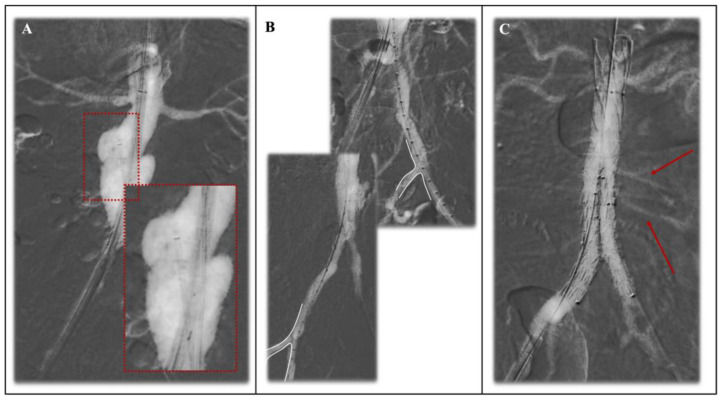
Endovascular aortic repair (EVAR) conducted with exclusive use of CO_2_-DSA for a penetrating aortic ulcer treatment with supra-aortic fixation endograft. (**A**) Pre-operative CO_2_-DSA performed with the use of 6F 55 cm introducer sheath (red box in details) after the navigation of the main body endograft in the aortic neck based on pre-operative bone markers and acquired with visualization of both renal arteries; (**B**) intraoperative CO_2_-DSA with good visualization of both left (upper) and right (lower) internal iliac arteries; (**C**) final CO_2_-DSA showing good result of the procedure and sealing of the treated penetrating aortic ulcer, with filling of the lumbar arteries (red arrows) without sign of type-II endoleak.

**Figure 5:**
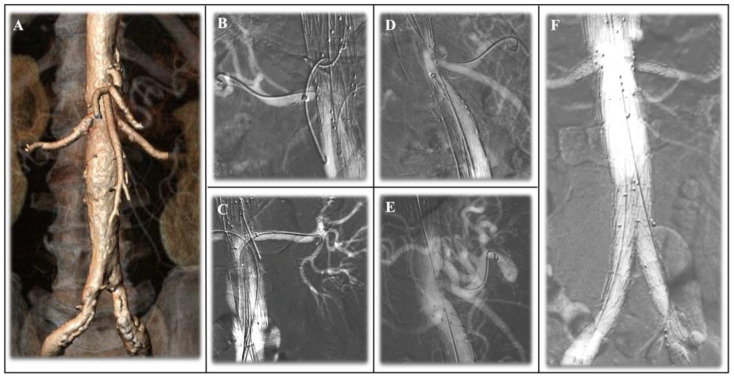
(**A**) Juxtarenal abdominal aortic aneurysm planned with a four fenestrated endovascular aortic repair (FEVAR) custom-made device for renal arteries (RAs), the superior mesenteric artery (SMA), and celiac artery (CA). (**B**) Details of CO_2_-DSA of the left RA after stent graft deployment. (**C**) Details of CO_2_-DSA of the right RA after stent graft deployment. (**D**) Details of CO_2_-DSA of the fenestrated SMA and (**E**) after fenestration for the CA. (**F**) Final CO_2_-DSA showing complete exclusion of the aneurysm, patency of the endograft, and the target visceral vessels.

Specifically, we included all series of patients treated with endovascular aortic repair (EVAR) for abdominal aortic aneurysms (Figure 4) and with fenestrated/branched endografts (F/B-EVAR) to treat complex abdominal aortic aneurysms (Figure 5) in which CO_2_-DSA was used as the main means of contrast. Results were analyzed separately for EVAR and F/B-EVAR procedures.

The primary outcomes were as follows: (1) impact of CO_2_-DSA in prevention of the postoperative renal function worsening (PO-RFW); (2) the accuracy of CO_2_-DSA in arterial visualization to adequately define proximal and distal landing zone. 

Postoperative renal function worsening (PO-RFW) was defined as either an increase in serum creatinine or reduction in eGFR. The secondary endpoint was the ability of CO_2_-DSA in visualizing intraoperative endoleaks.

Due to the narrative design of this review, we performed a descriptive presentation of the results of the included studies.

## 3. Results

### 3.1. CO_2_-DSA in EVAR: Techniques and Search of the Literature

The literature search resulted in 17 papers [14,17,18,19,20,21,22,23,24,25,26,27,28,29,30,31,32] reporting on the use of CO_2_ as contrast medium in standard endovascular repair, including 644 treated patients. Ten are comparative studies between the use of CO_2_ and ICM [14,18,20,21,22,23,24,25,27,31]. Among the CO_2_ cases, 372 (58%) were performed with zero iodinated contrast medium. Characteristics of the included studies are reported in Table 2. 

#### 3.1.1. Primary Endpoints

Eight of the included studies [14,18,21,22,23,24,25,27] focused on the impact of CO_2_-DSA in renal function protection during EVAR (Table 3). Four studies [14,18,21,25] reported a statistically lower rate of postoperative renal function worsening (PO-RFW) in patients undergoing CO_2_-EVAR compared to ICM-EVAR. Specifically, Criado et al. [25] reported a 12.7% greater decrease in the postoperative estimated glomerular filtration rate (eGFR) of patients treated with ICM-EVAR compared to the group treated with CO_2_ + additional ICM (*p* = 0.004) and a 10% greater decrease in eGFR when compared with the group of patients who had EVAR treated exclusively with CO_2_ (*p* = 0.042).

Another study [18] described significantly lower postoperative serum creatinine in the CO_2_-EVAR population compared to the ICM-EVAR group (*p* = 0.01), as well as lower mean postoperative eGFR decrease (*p* < 0.001).

Among the included studies, five [17,18,19,22,26] drew attention to the efficiency of arterial visualization in correctly identifying the proximal and distal landing zone during EVAR with CO_2_-DSA (Table 4). Visualization of both renal arteries used to individuate the proximal landing zone before graft deployment ranged between 53% and 100% [17,18,19,22,26]; visualization of both hypogastric arteries used to correctly identify the distal landing zone ranged between 94% and 100% [17,18,22].

#### 3.1.2. Secondary Endpoints

Six of the included studies [19,20,24,28,29,31] reported on the efficiency of endoleak (EL) detection during intraoperative angiographies performed with CO_2_. The main findings of these studies are summarized in Table 5.

Overall, studies reported no difference in endoleak detection between CO_2_-EVAR and ICM-EVAR. When considering low-flow type 2 endoleaks, three of the included studies [24,28,29] reported a poorer detection rate with intraoperative CO_2_-DSA. Another study [31] reported a higher sensitivity and specificity in type 2 endoleak detection compared to ICM-DSA. 

### 3.2. CO_2_-DSA in Fenestrated and Branched EVAR: Techniques and Search of the Literature

Only one study [33] on the use of CO_2_-DSA in complex endovascular aortic repair was retrieved, including 30 patients treated with fenestrated/branched endografts (F/B-EVAR). 

The study published by Gallitto et al. [33] on the use of CO_2_-DSA in F/B-EVAR included patients treated with combined CO_2_-DSA and fusion imaging. In this paper, the synergic role of the use of CO_2_-DSA is analyzed, together with the combination of fusion imaging based on the pre-operative imaging, with the aid of hybrid room facilities, in order to decrease ICM usage for endograft deployment and targeted visceral vessels’ visualization as much as possible. 

For the superior mesenteric artery and celiac trunk, in order to prevent specific damage, selective angiography is carried out using the standard procedure as a diagnostic treatment; automated CO_2_ injection is set at baseline level (650 mmHg pressure and 100 mL volume), and the CO_2_ is administered through the introducer sheath inside the aorta, with optimal visualization of the target artery (due to their anterior position). A different technique is applied for renal arteries, where selective angiographies are conducted inside the arteries and, in order to reduce potential damage, low pressure (80 mmHg) and low volume (60 mL) are set for the injector. 

Primary endpoints showed a significant lower serum creatinine level in patients treated with CO_2_-DSA compared to patients treated with standard ICM. No data were available on the arterial visualization and endoleak detection. No further experiences have yet been published in the literature. 

## 4. Discussion

This review summarizes the use of CO_2_ as a novel application in the endovascular treatment of the aortic pathologies in the abdominal segment as an alternative means of contrast to standard iodinated materials.

The results suggest that CO_2_-DSA might be an important technique for preventing postoperative renal function worsening in patients undergoing standard EVAR procedures, despite acceptable visualization of the target arteries. Notwithstanding, the evaluation of endoleaks and further applications in the field of complex AAA are instead still under debate, with promising use during F/B-EVAR.

Patients with chronic kidney disease (CKD) are at risk of developing post-contrast acute kidney injury (PC-AKI) [34]. Contrast-induced nephropathy (CIN) risk classification [13] has been proposed to recognize patients exposed to ICM who are at negligible risk of CIN (eGFR ≥ 45 mL/min/1.73 m^2^), intermediate risk of CIN (eGFR between 30 and 44 mL/min/1.73 m^2^), and high risk of CIN (eGFR < 30 mL/min/1.73 m^2^). In this latter case, it is suggested to avoid contrast-enhanced exams or procedures, whereas in cases of intermediate risk or high risk for CIN with unavoidable exposure to ICM, a prophylactic saline solution hydration and the minimization of contrast volume are recommended [10,11]. 

Hence, CO_2_’s main advantage in endovascular treatment, especially for patients with reduced renal function, is its lack of nephrotoxicity and anaphylactic response compared to ICM.

CO_2_ is a gas, and differing from ICM, it does not mix itself with the bloodstream, instead moving away the blood and placing itself in the superior portion of a large vessel like the abdominal aorta using the physical characteristic of CO_2_ called buoyancy. Thus, buoyancy is the upward force exerted by a fluid on an object that is partially or fully submerged in it. In the case of CO_2_, buoyancy is a result of its density, which is approximately 1.98 times that of air (Figure 1).

Moreover, the buoyant effect of CO_2_ contributes to reduced embolic risk during EVAR procedures. Unlike traditional iodinated contrast agents, which may dislodge plaque or thrombus, CO_2_ rises rapidly within the bloodstream, minimizing the risk of embolization. CO_2_ should be cautiously evaluated in patients with patent foramen ovale (PFO) or an atrial septal defect. Being about 400 times less viscous than iodinate contrast medium, CO_2_ can be injected through smaller introducers and small-bore needles. Carbon dioxide injection can be performed using diagnostic catheters or introducers. It is suggested to position the tip just above the territory of interest according to bone landmarks and computed tomography preoperative evaluation. Specifically, one study [30] compared the ability to detect the lowest renal artery with preimplantation CO_2_-DSA, showing significantly better image quality when using the femoral introducer rather than the pigtail (Figure 4A). This could be explained by the fact that the pigtail catheter, having multiple side holes, might cause higher gas dispersion. 

Most of the included studies focuses of the impact of CO_2_-DSA in protecting renal function compared with contrast medium. Specifically, Busutti et al. [14] described a significantly lower incidence of PO-RFW and higher eGFR values in immediate post-procedure controls as well as during the follow-up period. Moreover, one study also reported results in ruptured AAA, showing no significant increase in serum creatinine in patients treated with ICM compared to CO_2_ [27].

Some studies, focusing mainly on the use on manual CO_2_ for peripheral arterial disease, showed some issues regarding abdominal and leg pain as well as mesenteric ischemia issues [35]; however, in reported large series, no specific issues were found [18], testifying the safety of this procedure. Occasionally, mild bowel bloating has been observed, but came to a benign resolution [30]. 

Due to its characteristics, arterial visualization might represent a challenge. However, the literature shows acceptable results for renal arteries’ visualization before endograft deployment and excellent results for hypogastric arteries. Interestingly, Mascoli et al. [17] reported a higher failure rate in identification of the lowest renal artery in patients with a larger aneurysmal luminal volume using CO_2_-DSA. Another study [18] correlated inefficient lowest renal artery visualization using CO_2_-DSA with a short proximal neck, with a cutoff of 24 mm of neck length. In these cases, the positioning of the endograft at the level of the aortic neck (Figure 4A) can be helpful in reducing the space or the aortic lumen and therefore enabling the CO_2_ to more accurately fill the spaces, permitting better vessel visualization. 

Moreover, a multicenter study published in 2022 [30] compared lowest renal artery detection with CO_2_ preimplantation angiography with angiography performed through a pigtail catheter and femoral introducer, demonstrating significantly better image quality when using a femoral introducer rather than a pigtail (*p* = 0.008). With these tools combined, the 57% of the reported patients underwent a “zero-contrast procedure”, testifying the usefulness of this procedure and the rarer need for ICM. At the same time, these results represent the initial learning curve with these devices, with a future trend of a higher number of cases with 100% CO_2_ in experienced and dedicated aortic centers. 

Another discussed topic is endoleak detection, especially when focusing on type II endoleaks. Studies show an overall comparable rate of detection of type I-III endoleaks between CO_2_ and ICM-DSA. However, the results on endoleak detection with CO_2_-DSA are discordant. Specifically, Quaglino et al. [24] reported a more accurate type II EL detection with CO_2_-DSA compared to ICM-DSA. Sueyoshi et al. [28] reported a sensitivity of 0.50 and a specificity of 1.00 for CO_2_-DSA compared to ICM intraoperative angiographies, showing that of the type II endoleaks detected on ICM-DSA but not on CO_2_- DSA, none progressed to persistent type II EL during follow up. Another paper published by Mascoli et al. [31] showed that CO_2_-DSA has an high sensitivity and specificity for high-flow and low-flow endoleaks and higher agreement with CEUS if compared with ICM-A for type II endoleak detection.

At the present time, only one study has been published in literature focusing on the potential benefit of combined carbon dioxide angiographies in complex endovascular aortic repair. Gallitto et al. [33] aimed to evaluate the effectiveness of combined carbon dioxide automated angiography and fusion imaging in preserving perioperative renal function in patients undergoing FEVAR. The study included 30 patients who underwent fenestrated endografting with either combined CO_2_-DSA and fusion imaging standard angiography. The results showed significant lower serum creatinine levels for patients with the combination of the two mechanisms compared to those who underwent standard angiography. The use of this imaging modality may help to reduce the risk of perioperative renal dysfunction and improve patient outcomes. As such, it is an important tool that should be considered in the perioperative management of patients undergoing fenestrated endografting. At the same time, more studies are required to confirm these findings, and the use of CO_2_-DSA should be considered specifically for fenestrated repair of complex AAA (Figure 5) because the repair is conducted below the diaphragm; its use in branched endografting and for complex/extended thoraco-abdominal aortic aneurysm is still limited, as confirmed by the lack of data on these procedures in the latest endovascular series recently available both in the elective and urgent setting [36,37].

Of paramount importance is the setting in which these procedures are conducted. As stated, the use of a dedicated automated injector has revolutionized the use of CO_2_-DSA during EVAR, allowing standardization, efficiency and safety [38,39]. At the same time, the use of a hybrid room with fusion imaging facilities may help in reducing the number of angiographies needed for precise endograft deployment and improving arterial detection both in the standard setting and, more importantly, in complex repair for the visualization of the renal and mesenteric arteries, in which precise fusion imaging is known to speed up canulation procedures [40]. In this complex field, if not investigated specifically by this narrative review, the synchronous use of intravascular ultrasound (IVUS) is a useful technology for reducing selective angiographies and detecting precise stent graft apposition or damage to target arteries without the use of additional means of contrast [41]. Strong collaboration with radiographers to customize an appropriate CO_2_-DSA mask for post-processing work will be essential for optimizing the performance of these procedures [38,42].

In order to reduce the amount of radiation and ICM exposure for this frail patient population with impaired renal function and risk of PC-AKI, follow-up in should attentively adhere to the most recent 2024 ESVS Guidelines [1], where after the first-year CTA, in cases of suspicious signs of EVAR failure, follow-up can be performed after early imaging using duplex ultrasound only and using a CTA after 5 years. A similar application after the first year standard CTA follow-up can be reserved for selected patients undergoing complex AAA repair. 

This review has some limitations, one of which is its narrative nature. This choice was driven by the intention not to analyze a single outcome (e.g., renal function worsening) but to provide an overview of the role of CO_2_ in aortic endovascular procedures. At the same time, such comprehensive studies can be difficult at the present stage, since the modalities and protocols present in the literature, as well as endpoints, are still too heterogenous to perform such a specific analysis. Future studies focusing on the use of CO_2_-DSA in the treatment of EVAR should be consistent in terms of mechanism and definitions, and a general consensus on the use of standardized protocols for CO_2_ volume and pressure, the use of RIFLE classification for renal function worsening, the visualization of the renal arteries for standard EVAR and reno-visceral vessels for complex technologies as well as hypogastric arteries, the rate of endoleak detection intraoperatively and its relevance compared to postoperative follow-up and clinical aneurysmal sac evolution, and the rates of CO_2_-DSA-related complications should be defined. All these aspects together may represent the basis for large comparative/randomized studies and permit low-heterogeneity reviews and metanalyses with the final aim of standardizing this widespread technique, driving some recommendations in vascular guidelines. 

A final remark concerns costs: at the present time, it is not possible to perform an analysis of this aspect. From a theoretical point of view, CO_2_ as a mean of contrast is cheaper than ICM; at the same time, though, the cost of a dedicated automated injector should be balanced by the presence of the standard ICM injector used in everyday endovascular activities. At the same time, the cost of training and specific radiological masks should be considered; however, this extra cost should be mitigated by the clinical expenses of a reduced number of patients experiencing PC-AKI and associated prolonged hospitalization (and the resources this demands). However, at the moment, few data are available to correctly address this topic. 

## 5. Conclusions

The use of carbon dioxide in endovascular aortic repair (EVAR) represents a significant advancement in the field, offering a promising alternative to iodinated contrast medium with benefits especially for patients with impaired renal function. The summary of the results after this narrative review demonstrates the effectiveness of CO_2_-DSA in minimizing postoperative renal function worsening in an increasing number of “zero-contrast” EVAR procedures. Arterial visualization is possible considering the physical characteristics of CO_2_ together with some tips facilitating the procedure, whereas the role of CO_2_ in endoleak detection is still debatable; other specific endpoints could therefore be subject to larger studies and more systematic analyses. The use of CO_2_-DSA in complex abdominal repair is the next step for future studies, with first experiences showing good results in renal function protection; it may be accompanied by other diagnostic tools in order to reduce amount of radiation and ICM administered both during the procedure and during the follow-up period. 

## Figures and Tables

**Table 1:** Summary of the main characteristics, advantages, disadvantages, and systems comparing iodinated contrast media and cardon dioxide contrast media.

	Iodinate Contrast Media	Carbon Dioxide Contrast Media
Physical characteristics	High blood miscibility	High buoyancy Low viscosity
Advantages	Good visualization of all visceral vessels	No nephrotoxicity No hepatotoxicity Nonallergic
Disadvantages	Nephrotoxic Hepatotoxic Allergenic	Prohibited for use above the diaphragm Difficult visualization of visceral vessels originating from the posterior aortic wall Higher radiation exposure
Injector system	Manual Automated injectors	Manual Dedicated automated injectors

**Table 2:** Characteristics of the included studies on the use of CO_2_-DSA during EVAR procedures.

Author et al.	Year	Study Design	Setting	No. of CO_2_ Patients	Comparative Study	No. Control ICM Patients	Zero-Contrast Cases, No.	Article Focus
Chao et al. [20]	2007	R	Los Angeles, California	16	yes	84	3	Endoleak detection with CO_2_ DSA angiography
AD. Lee et al. [19]	2010	R	Kingswood, Australia	17	no	0	0	Impact of CO_2_-DSA in renal function protection and intraoperative arterial visualization
Knipp et al. [27]	2010	R	Ann Arbor, Michigan	4	yes	7	3	Impact of CO_2_-DSA in renal function protection in ruptured AAA
Criado et al. [25]	2012	R	Ann Arbor, Michigan, USA	114	yes	0	72	Comparison of renal function protection between CO_2_ and CO_2_+ICM angiographies
Huang et al. [29]	2012	P	Los Angeles, CA	76	no	0	76	Endoleak detection with CO_2_ DSA angiography
Sueyoshi et al. [28]	2015	P	Sakamoto, Japan	40	no	0	40	Endoleak detection with CO_2_ DSA angiography
Mendes et al. [22]	2017	RCT	San Paolo, Brazil	16	yes	16	6	Impact of CO_2_-DSA in renal function protection
De Angelis et al. [26]	2017	R	Milano, Italy	17	no	0	16	Efficacy of CO_2_-DSA in arterial visualization and graft deployment
Takeuchi et al. [23]	2018	R	Yamaguchi, Japan	30	yes	351	0	Impact of CO_2_-DSA in renal function protection
Mascoli et al. [17]	2018	R	Bologna, Italy	31	no	0	31	Efficacy of CO_2_-DSA in arterial visualization and graft deployment
Mascoli et al. [31]	2018	R	Bologna, Italy	21	yes	0	16	Type II endoleak detection
Vacirca et al. [18]	2022	R	Bologna, Italy	72	yes	249	16	Impact of CO_2_-DSA in renal function protection and arterial detection
Unal et al. [21]	2023	R	Ankara, Turkey	34	yes	34	0	Impact of CO_2_-DSA in renal function protection
Busutti et al. [14]	2023	R	Bologna, Italy	22	yes	22	5	Impact of CO_2_-DSA in renal function protection
Quaglino et al. [24]	2023	R	Turin, Italy	52	yes	49	52	Impact of CO_2_-DSA in renal function protection and endoleak detection
Vacirca et al. [30]	2023	P	Multicenter	65	no	0	19	Comparison of arterial visualization before, during, and after graft deployment
Esposito et al. [32]	2023	R	Florence, Italy	17	no	0	17	Evaluation of feasibility and safety of a “zero-contrast” approach in patients with CKD.
TOTAL				644		812	372	

CO_2_ = carbon dioxide; ICM = iodinated contrast media; DSA = digital subtraction angiography; CKD = chronic kidney disease. R = retrospective; P = prospective RCT = randomized control trial.

**Table 3:** Primary endpoint with the evaluation of the impact of CO_2_-DSA in the prevention of postoperative renal function worsening (PO-RFW) after EVAR.

Author et al., Year	No. of CO_2_ Patients	No. of Control ICM Patients	Matched Cohorts	Adjunctive ICM Used for CO_2_ (SD/Range) mL	PO-RFW *n* (%)/in CO_2_-EVAR	PO-RFW *n* (%) ICM-EVAR	*p* Value*	Definition of PO-RFW	ΔsCr in CO_2_-EVAR (SD/Range) micrommol/L	ΔsCr in ICM-EVAR (SD/Range) micrommol/L	*p* Value°
Knipp et al., 2010 [27]	4	7	no	0	-	-	-	-	0.25 ± 0.19	0.58 ± 0.25	0.066
Criado et al., 2012 [25]	114	0	-	37 (3.7)	-	-	-	-	-	-	-
Mendes et al., 2017 [22]	16	16	yes	5.5 (0–15)	-	-	-	-	11.1 (3.1–22.6)	11.7 (5.1–19.5)	0.80
Takeuchi et al., 2018 [23]	30	351	no	18 (15)	10 (1)	1 (3.3)	0.93	RIFLE classification	-	-	-
Vacirca et al., 2022 [18]	72	249	no	52.8 (6.1)	-	-	-	-	0.08 ± 0.04	0.17 ± 0.09	0.01
Quaglino et al., 2023 [24]	52	49	no	0	-	-	-	-	1.1 (0.8–1.3)	0.98 (0.85–1.2)	0.401
Unal et al., 2023 [21]	34	34	yes	4 (8)	2.8	23.5	0.027	25% increase in sCr or a 0.5 mg/dL increase sCr within 48 h	-	-	-
Busutti et al., 2023 [14]	22	22	yes	49.5 (35)	9	27	<0.05	KDIGO	-	-	-

CO_2_ = carbon dioxide; ICM = iodinated mean of contrast; SD = standard deviation; mL = milliliters; PO-RFW = postoperative renal function worsening; Delta Cr = difference in postoperative minus pre-operative creatinine, calculated in micromoles over liter; *p* value* significant if <0.05 in studies comparing CO_2_-EVAR and ICM-EVAR based on reported PO-RFW; *p* value° for studies comparing differences in delta creatinine with CO_2_-EVAR vs. ICM-EVAR.

**Table 4:** Primary endpoint of the reported arteries targeted for visualization with the use of CO_2_-DSA during EVAR procedures.

Author et al., Year	*n* Patients	Both Renal Arteries *n* (%)	Aortic Bifurcation *n* (%)	Hypogastric Arteries *n* (%)	Ipsilateral Iliac Artery *n* (%)	Contralateral Iliac Artery *n* (%)
AD. Lee et al., 2010 [19]	17	9 (53)	17 (100)	-	17 (100)	17 (100)
Mendes et al., 2017 [22]	16	16 (100)	16 (100)	15 (94)	16 (100)	16 (100)
De Angelis et al. 2017 [26]	17	17 (100)	17 (100)	-	-	-
Mascoli et al., 2018 [17]	31	19 (61)	-	31 (100)	-	-
Vacirca et al., 2022 [18]	72	50 (69)	-	72 (100)	-	-

**Table 5:** Secondary endpoints of the rate of endoleak detection with CO_2_-DSA during EVAR, with the main findings of the studies summarized.

Author et al., Year	Endoleak Detection with CO_2_-DSA: Main Findings
Chao et al., 2007 [20]	No difference in endoleak detection between CO_2_ and ICM angiographies
AD. Lee et al., 2010 [19]	No difference in endoleak detection between CO_2_ and ICM angiographies
Huang et al., 2012 [29]	CO_2_-DSA has poor sensitivity and poor positive predictive value in the detection of ELII.
Sueyoshi et al., 2015 [28]	Lower endoleak detection with CO_2_-DSA (40%; 16/40) compared to ICM-DSA (68%;27/40). No difference in detection of type I–III EL. Among the type II EL detected on ICM-DSA but not on CO_2_-DSA, none progressed to persistent type II EL.
Mascoli et al., 2018 [31]	Type II EL detection with CO_2_-DSA has a higher agreement with CEUS detection if compared to ICM-DSA
Quaglino et al., 2023 [24]	Lower intraoperative type 2 EL detection rate (14.3%; 7/49) in the ICM group compared to CO_2_ group (25%; 13/52) (*p* = 0.2).
